# Seasonal Variations in Physical Fitness and Performance Indices of Elite Soccer Players

**DOI:** 10.3390/sports6010014

**Published:** 2018-02-13

**Authors:** Yoav Meckel, Ofer Doron, Eyal Eliakim, Alon Eliakim

**Affiliations:** 1 The Academic College at Wingate, Wingate Institute, Netanya 4290200, Israel; oferon1@gmail.com (O.D.); eyale0301@gmail.com (E.E.); Eliakim.Alon@clalit.org.il (A.E.); 2 Child Health and Sports Center, Pediatric Department, Meir Medical Center, Tel-Aviv University, Sackler School of Medicine, Tel-Aviv 6100000, Israel

**Keywords:** training, aerobic and anaerobic capabilities, physical testing

## Abstract

The aim of the study was to investigate seasonal variations in fitness and performance indices of professional male soccer players. Eighteen professional male soccer players (age range 22–32 years) completed three similar sets of tests at three stages of the season: before preseason; after preseason and the middle of the competitive in-season. A significant decrease in body mass and percent fat was found during the preseason. A significant improvement (*p* < 0.05) was found in the vertical jump (preseason: 37.0 ± 5.3, post-preseason: 39.0 ± 4.8, mid-season: 40.3 ± 5.5 cm), the 4 × 10-m agility test (preseason: 8.1 ± 0.2, post-preseason: 7.9 ± 0.2, mid-season: 8.1 ± 0.3 s), flexibility (preseason: 45.2 ± 8.8, post-preseason: 48.2 ± 7.0, mid-season: 49.9 ± 6.9 cm) and aerobic capacity (preseason: 52.7 ± 6.6, post-preseason: 56.4 ± 6.0, mid-season: 57.4 ± 5.4 mL/kg/min) during preseason, with no further change during mid-season. Repeated sprint test (RST) (6 × 30-m) performance indices showed significant deterioration (*p* < 0.05) in ideal sprint time (IS; preseason: 21.8 ± 1.0, post-preseason: 23.0 ± 0.8, mid-season: 23.2 ± 0.8 s) and total sprint time (TS; preseason: 22.5 ± 0.7, post-preseason: 23.5 ± 0.6, mid-season: 23.8 ± 0.6 s) during preseason, with no further changes during mid-season. However, performance decrement (PD) significantly decreased during the preseason with no change during mid-season. The findings suggest that while power training was probably responsible for the anaerobic fitness improvement, the high-volume training led to improvement in aerobic fitness during the preseason. However, the low-intensity aerobic-type training, coupled with the high total training load, may have led to fatigue and decreases in IS and TS during the preseason.

## 1. Introduction

In modern professional soccer leagues, players are in active training for around 10 to 11 months. While the preseason period is relatively short and lasts only about two months, the competitive season is stretched over a long period of 8 to 9 months. During the competitive phase, teams are playing every weekend and reach a total of 30 league games and 1–6 Cup games. Once the competitive season is completed, the players usually move into a resting stage of about four to six weeks—an off-season period—before starting a new preseason.

Although the player’s physical fitness is only one of several variables, it plays a key factor in a soccer team’s success [1,2]. However, since players are subject to changes in playing time, training intensity, injuries, nutrition and playing position, it is essential to monitor their physical fitness and performance capabilities throughout the season. The outcome of such procedures may provide coaches with necessary information on whether to carry on with the pre-planned training program or to make some applicable changes.

In general, the predominant metabolic pathway in professional soccer is aerobic, and that higher maximal oxygen consumption (VO_2_ max) is significantly correlated with key game performance indices, such as total distance and high-intensity running distance covered by the players, number of sprints and number of contacts with the ball during the game [3,4]. Nevertheless, Reilly et al. [5] have found that anaerobic performance capabilities, such as sprinting, jumping and changing directions, are essential to the soccer match outcome. Therefore, the level of the aerobic and anaerobic fitness domains, along with a balance between the two, are essential for soccer performance and need to be evaluated continuously throughout the season [6].

The available literature regarding seasonal variation in physical fitness among soccer players demonstrates some inconsistent evidence. For example, while some studies reported significant improvement in speed and power performances throughout the preseason period, and a further improvement until the middle or the end of the competitive season [7,8], others reported improvement during the preseason with no further changes during the competitive season [5,9,10]. In the same line, while aerobic fitness was found to be significantly improved during the preseason phase in all relevant studies, some of them reported stagnation in this variable throughout the competitive season [8,11], whereas others reported increases [12,13] or decreases [14,15] during that period. One reason for these inconsistencies may be the differences between the participants since some studies tested professional players [9,15,16] while others examined semi-professional adult [12] or young [7,13] players. Naturally, the differences in volume and intensity of training may have induced differences in fitness status and performance capabilities in each of these populations throughout the season. It is also noted that research has focused on certain fitness components and usually does not present a more global view of the player’s physical condition throughout the season.

One evaluation measure that seems to be relevant to the activity pattern in soccer, and therefore has become very popular among soccer investigators, is the repeated sprint test (RST) [17,18]. This procedure determines several performance indices: ideal sprint time, total sprint time and performance decrement, all of which are relevant to the players’ challenges during soccer games. Moreover, performance indices of the RST were significantly correlated with the aerobic fitness of soccer players [19]. Surprisingly, to the best of our knowledge, the RST and its relevant performance indices have not been used to monitor professional soccer player’s fitness variations throughout the season. The aim of the present study, therefore, was to investigate seasonal variations in relevant aerobic and anaerobic fitness and performance indices of professional soccer players.

## 2. Materials and Methods

### 2.1. Participants

Twenty-three trained male soccer players (age range 22–32), members of a first division league team in Israel, began the study. However, five of the 23 players were excluded from the final analysis. The 21% drop-out rate occurred due to missed follow-up tests, injuries, or absence from more than 10% of the training sessions. Thus, only 18 participants were included in the final analysis. All the players had at least three years of professional-level competitive and training experience. The players were informed of the study’s requirements, benefits and risks before giving their written informed consent. The study was approved by the institutional research ethics committee. The anthropometrical characteristics of the players, at the different phases of the season, are presented in Table 1. Standard calibrated scales and stadiometers (Seca, 707, Germany) were used to determine body mass and height. Skinfold measurements at four sites (triceps, biceps, sub-scapular and supra-iliac) were taken by an experienced technician.

### 2.2. Procedures

Testing was performed in the following three key stages of the season: Test 1 (beginning of July): before the start of preseason training; Test 2 (end of August): upon completion of preseason training; Test 3 (end of January): middle of the competitive season. Testing procedures at the three time points were conducted on two non-consecutive days (Monday and Thursday). Coaches were asked to avoid an intense workload on the day prior to testing. All tests were conducted at the same time of the day (late afternoon), under comfortable weather conditions, on the same soccer pitch, and with the same technicians. All tests were performed about four hours after lunch and 30 min after drinking 500 cc of water. Testing at all stages was conducted using the same procedures and measurement devices. Participants were familiar with the testing procedures, as they had performed them on previous occasions. Prior to each set of tests (excluding the multistage laboratory aerobic test), participants performed a standard warm-up that included 8 min of jogging followed by 10 min of stretching exercises, two 20-m sprints, some jumping and specific coordination drills. About 20–25 min and 2–4 min separated the different tests and trials, respectively, on each testing day. Running times were recorded using a photoelectric cell timing system (Alge-Timing Electronic, Vienna, Austria) with an accuracy of 0.001 s, linked to a digital chronoscope. A standing start, with the front foot, placed 30 cm behind the timing lights, was used for all sprints. Timing was initiated when the subject broke the light beam. Timing lights were set at 90 cm height directed to the player’s upper thigh area.

Fitness and performance measurements in each stage included the following tests: sit and reach; 4 × 10 m run; countermovement vertical jump (CMJ); a multistage aerobic test and an RST. The rationale for the testing procedure’s selection was based on their relevancy to soccer. In this respect, the RST use was especially relevant, as it resembles the activity pattern of the game. This testing procedure is used by most soccer coaches to follow their players’ fitness status. High test–retest reliability (0.80–0.95) of most of the tests used in this study was reported previously [4,20,21].

### 2.3. Testing Protocols

On the first testing day of each testing week, the participants performed the following three procedures.

#### 2.3.1. Sit and Reach—Flexibility Test

Participants sat with their legs extended on the floor. Their feet were placed against a box with a yardstick attached to it. Participants placed their hands one on top of the other and slowly extended them forward, and then held them at the maximum stretch point for 3 s [4]. The distance reached on the yardstick was recorded. Three attempts were made and the longest distance on the yardstick was recorded.

#### 2.3.2. CMJ—Power Test

Vertical jump height was measured using the countermovement technique. Participants began in an erect standing position, moved into a semi-squat position and then used a vigorous double-arm swing before jumping as high as possible. All jumps were performed on a 60 cm × 40 cm force platform (Kistler 9286, Kistler Instrument Corporation, Amherst, MA, USA) connected to a digital timer that recorded the flight time of all jumps. The flight time was used to calculate the body’s center of gravity height change (t_flight_^2^·g)/8. Three trials were performed, and the highest jump achieved was recorded.

#### 2.3.3. A Multistage Running Test—Aerobic Measurements

Participants performed incremental running on a motor-driven treadmill (PPS MED, Woodway, Weil am Rhein, Germany) in order to detect their maximal aerobic power (VO_2_ max) and anaerobic threshold (AT). All participants were familiar with treadmill running. For a warm-up, the participants ran on the treadmill for 8 min at a speed of 8–8.5 km/h, which was followed by 10 min of stretching drills. For the actual test, the treadmill’s initial speed was 9 km/h with a 1% grade. The speed was increased by 1 km/h every minute until volitional exhaustion occurred. The grade was maintained at 1% throughout the entire test. The test was considered maximal if at least two of the following criteria were fulfilled: plateau in VO_2_, heart rate (HR) >95% of the age-predicted maximum, or a respiratory exchange ratio (RER) greater than 1.15. Oxygen consumption (VO_2_), pulmonary ventilation (VE), carbon dioxide output (VCO_2_), respiratory exchange ratio (RER) and the ventilatory anaerobic threshold (AT) were monitored breath-by-breath, using an automated on-line metabolic analysis system (CPET K-4, Cosmed Quark, Rome, Italy). Polar Accurex monitors (Polar Accurex Plus, Polar Electro, Woodbury, NY, USA) were used to follow HR responses. The metabolic cart was calibrated before each test according to the manufacturer’s instructions. The criterion for the non-invasive (ventilatory) determination of the gas anaerobic threshold was the modified V-slope method, in which VCO_2_ is plotted against VO_2_ (4). The AT was defined as the last value prior to the departing of VCO_2_ versus VO_2_ slope from linearity. The AT was determined visually in a blinded manner by two investigators. A third investigator was consulted when the two investigators did not agree on threshold placement. Running speed (km/h) at the anaerobic threshold level was recorded.

On the second day of each testing week, participants performed the following two tests:

#### 2.3.4. 4 × 10-m Run—Agility Test

The agility test used was the 4 × 10-m back and forth shuttle run. The participant began at the baseline and ran at maximal speed to a marked line that was located 10 m ahead of him. He then turned and ran back to the baseline, turned again and performed the same back and forth run. This procedure was performed twice, and the faster time of the two trials was recorded.

#### 2.3.5. RST—Anaerobic Performance Indices

Participants performed a series of all-out short sprints in order to identify the player’s anaerobic capabilities. The RST protocol consisted of a 30 m run performed six times with 30 s recovery between runs. A 30-m all-out sprint, which was performed following the warm-up, was used as the criterion score during the subsequent RST. In the first sprint of each test, participants were required to achieve at least 95% of their criterion score. If 95% of the criterion score was not achieved, the participant was required to start the RST again. During the recovery between sprints, the participants tapered down from the sprint just completed and walked back to the next start point. Two sets of timing gates were used, working in the opposite direction, to allow subjects to start the next run from the same end at which they finished the preceding sprint, thus eliminating the need to hurry back to a common start point. An experimenter was placed at each end of the track to give strong verbal encouragement to each subject during each sprint. Participants were instructed prior to the test to produce their maximal effort for each sprint and to avoid pacing themselves. The three performance indices for the RST were the ideal (fastest) 30 m sprint time (IS), the total sprint time (TS) of the 6 sprints, and the performance decrement (PD) during the test. IS was used as an indication of maximal speed and was calculated as the best 30 m sprint time multiplied by six. TS was used as an indication of speed endurance and was calculated by the summation of all the sprint times. The PD was used as an indication of fatigue and was calculated as [(TS/IS) × 100] − 100. The test–retest reliability of running the RST is 0.942 for TS, and 0.75 for the PD [22].

### 2.4. Training Protocols

The preseason phase lasted eight weeks—from the beginning of July to the end of August—while the in-season period lasted 8.5 months—from the beginning of September to the middle of May. A typical weekly schedule of the pre- and the in-season training program is presented in Table 2. As can be seen, the total volume and the number of training sessions during the preseason is much higher than in the in-season phase (9 vs. 5 sessions), including strength and endurance-type training that was performed almost solely in the preseason. The strength training in the preseason included a weight lifting program (3–5 sets of 6–12 repetitions in 60–90% of one repetition maximum). At the advanced stages of the preseason phase, dynamic power training sessions, including plyometric jumps were incorporated (1–2 per week) instead of some of the weight lifting sessions. The endurance training in the preseason included continuous low-intensity running of 45–60 min at a constant or varied pace, and moderate and high-intensity interval training with a short rest between runs. Although small-sided games appear as a separate issue, they can also be classified as endurance-type training, similar to interval training in terms of physiological load, incorporated with soccer-specific technical skill. While this training method was used in the pre- and the in-season phases, it was usually performed at a higher intensity and at shorter distances during the in-season compared to the preseason phase.

### 2.5. Statistical Analysis

A two-way repeated-measures ANOVA was used to compare changes in fitness characteristics and performance indices throughout the soccer season using the statistical package for the social sciences (SPSS), version 20.0. Data are presented as mean ± SD. The significance level was set at *p* ≤ 0.05.

## 3. Results

**Figure 1:**
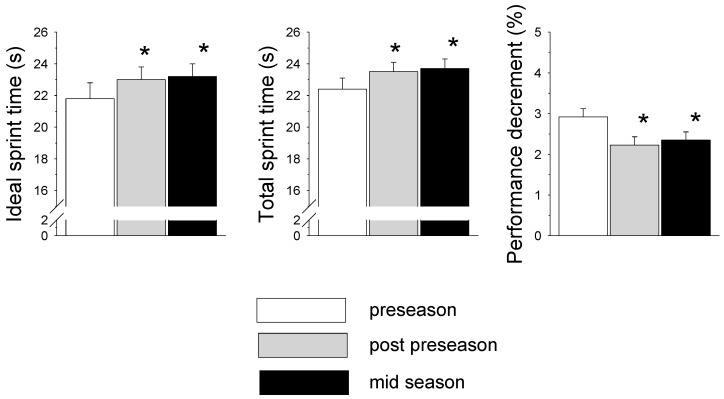
Changes in repeated sprint test (RST) indices of the soccer players throughout the different stages of the season (* *p* < 0.05 from preseason).

**Figure 2:**
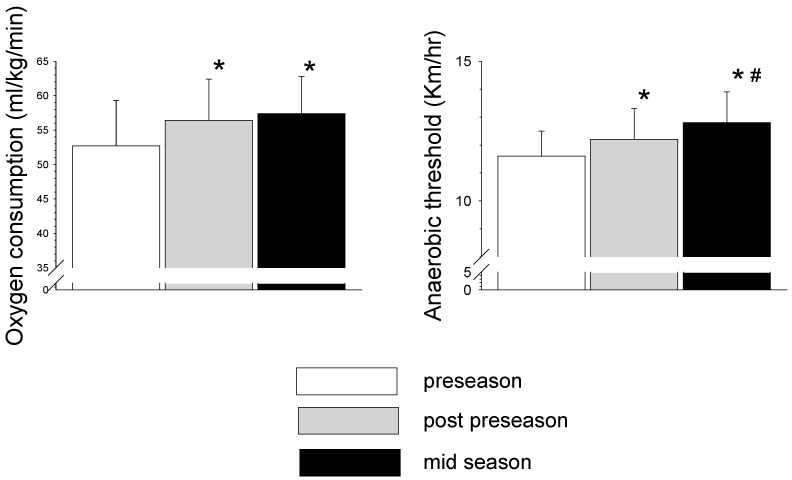
Changes in peak VO_2_ and anaerobic threshold (AT) running speed of the soccer players throughout the different stages of the season (* *p* < 0.05 from preseason; # *p* < 0.05 significant differences from post-preseason).

Anthropometric and fitness characteristics of the players are presented in Table 1. There was a significant decrease (*p* < 0.05) in body mass and percent body fat in post-preseason and mid-season measurements compared to the preseason measurements. There was a significant improvement (*p* < 0.05) in flexibility determined by the sit and reach test and vertical jump in post-preseason and mid-season measurements compared to the preseason measurements (Table 1). There was a significant improvement (*p* < 0.05) in agility determined by the 4 × 10 m test in post-preseason compared to the preseason measurements. (Table 1). Changes in indices of the repeated sprint test (RST) are presented in Figure 1. IS and TS were significantly higher (*p* < 0.05) in post-preseason and mid-season compared to pre-season values. In contrast, the PD decreased significantly (*p* < 0.05) in post-preseason compared to preseason measurements. Changes in peak oxygen consumption (peak VO_2_) and anaerobic threshold (AT) are presented in Figure 2. Peak VO_2_ improved significantly (*p* < 0.05) in post-preseason and mid-season compared to the preseason level. AT improved significantly (*p* < 0.05) between preseason to post-preseason and continued to improve significantly (*p* < 0.05) between post-preseason to mid-season.

## 4. Discussion

The findings of the study demonstrated significant improvement in the vertical jump, the 4 × 10 m test and flexibility during the preseason, with no further change in the mid-season. Similarly, the indices of aerobic fitness—VO_2_ max and AT—were significantly improved during the preseason, with further significant improvement in AT in mid-season compared to post-preseason measures. However, RST indices showed that while the PD was improved during the preseason period, IS and TS times became significantly slower during the preseason period.

The decrease in percent body fat and body mass during the preseason phase in the present study is in agreement with previous studies on professional soccer players [10,16,23,24]. The changes in body mass and percent body fat may be the result of the high training volume, especially the prolonged low-intensity endurance activity coupled with the higher intensity of long interval training and friendly games in this phase. This is consistent with the studies of Reilly [5] and Bangsbo [2] who stated that the significant decrease in body mass and fat percent during the soccer preseason is largely attributed to the large use of fat stores as energy for these activities, and that match play places a high demand on the aerobic energy system (90%) and causes a large energy expenditure.

The significant improvement in anaerobic capabilities such as explosive power and agility (measured by the vertical jump and the 4 × 10 m test, respectively) among professional soccer players during the preseason period is also in line with previous studies [12,13,14]. This improvement in agility was attributed mainly to the high-intensity nature of game-related training, such as small-sided games and friendly games [12,13]. This observation was supported by Mercer et al. [23] and Reilly [5], who reported that the frequent changes of directions (approximately every three seconds) in small-sided games help to increase motor coordination, which is essential for improved agility performance. Significant improvement in changes of direction was also found during the preseason in young players, with further improvement in this performance during the competitive season [13].

In line with our findings, Wisloff et al. [25] Hoff and Helgerud [26] concluded that vertical jump performance tends to improve in professional soccer players when weight training and plyometric training is performed frequently during the preseason phase. Significant improvement in vertical jump during the preseason phase, with no further improvement during the in-season phase, was also reported by Fessi et al. [14] and Wong et al. [27] following a combined training program of high-load muscular strength training and high-intensity interval training. These changes in power are consistent with Reilly [5], who demonstrated an improvement in vertical jump performance during the preseason of professional players with no further change during the competitive season. It is also possible that the improvement in anaerobic activities such as vertical jump and 4 × 10 m running in the present study reflects an improvement in specific motor skills that were practiced during soccer-specific technique training sessions during the preseason (see Table 2). The lack of vertical jump improvement during the competitive season in the present study may reflect the elimination of strength training during the competitive season. A stagnation in power performance was previously found during the competitive season in other studies on professional soccer players [9,10]. The fact that power performance did not decrease during the competitive season, even though strength training was cut off, confirms previous findings showing that muscle strength and power remained stable providing that previous training included specific explosive strength exercises [28].

The significant improvement in flexibility found during the preseason in the present study is in line with previous studies that found such improvement among professional [1] and semi-professional [12] soccer players. However, these findings are in contrast with others that demonstrated no changes in flexibility among professional players during the preseason [23,29]. The improvement in flexibility in the present study may be the result of the stretching exercises performed throughout the preseason period (Table 2).

One of the requirements in professional soccer is the ability of players to repeatedly perform short sprints throughout the match [2]. Surprisingly, to the best of our knowledge, variation in this ability as measured by RST performance indices (i.e., IS, TS and PD) has never been monitored among professional players throughout the season. The present findings indicated a significant decrease in IS and TS performance from the preseason to the post-preseason and to the mid-season. A number of factors may be involved in this reduced performance. First, it may reflect differences in the player’s nature of training and physical load during these phases. As described earlier, a major part of training during the preseason focuses on prolonged low-intensity endurance activity (Table 2). Although such training may induce cardiovascular and aerobic improvement, it may also depress neuromuscular function related to power and sprint performances [30]. In addition, the players in the present study performed an average of nine weekly training sessions during the preseason, including days that included two training sessions. It is possible that the high number of sessions, along with the high volume of endurance-type training, may have induced fatigue, causing a reduction in IS and TS during the preseason [31,32]. Although such fatigue was not noted in single short tasks such as the vertical jump or the 4 × 10 m agility test in the preseason, it seems to be a significant factor in anaerobic multiple-sprint tasks, such as the 6 × 30 m RST used in the present study. Given the game’s characteristics and requirements, the reduced IS and TS performance may raise concern among coaches and players. Therefore, the accumulated training load during the preseason should probably be monitored in order to try and reduce the player’s symptoms of fatigue. Most previous studies have shown improved speed during the preseason [6,10,14] or unchanged speed during the competitive season [13]; others have found reduced speed during the pre- or in-season in young players [12,33]. The investigators argued that one possible explanation for such a decrease may be the aerobic nature of training (in the preseason phase) or the accumulated load of training and games (in the competitive phase).

In contrast to the IS and TS findings, the PD improved significantly during the preseason in the present study (Figure 1). This improvement may reflect the player’s ability to recover faster between sprints and maintain speed throughout the RST. Tomlin and Wenger [34] suggested that high aerobic fitness is a prerequisite for increased anaerobic performance during sustained intermittent activities. This relies on the fact that creatine phosphate (CP) re-synthesis occurs primarily by oxidative processes [35]. Moreover, it was found that the involvement of aerobic energy sources in intermittent-type activities such as soccer increases as the amount of work and fatigue level is increased [19]. In light of this, the present study’s findings of a significant improvement in aerobic indices such as VO_2_ max and AT may explain the significant improvement in the RST PD in the post-preseason and mid-season compared with the beginning of the preseason (see Figure 2).

The significant increase in aerobic fitness (VO_2_ max and AT) in the present study is in agreement with previous reports of professional [14,16] and trained [13] young soccer players. A significant increase was also noted in the maximal aerobic speed of top-level players in first division European leagues during the preseason [3,20]. This increase was usually observed during the preseason and remained unchanged during the in-season competitive period. Kalapotharakos et al. [16] found a significant improvement in VO_2_ max (4.5%) and AT (10.8%) after the preseason training of elite players which remained unchanged during the competitive period. As much as a 24% increase in VO_2_ max was reported in young soccer players following preseason training [24]. Also, an 8% increase in AT was found in professional British players during the preseason period [36]. These aerobic indices remained unchanged during the competitive season. The improvement seen in aerobic fitness in previous studies and in the present study during the preseason was likely the result of the relatively high aerobic-type training volume performed during this phase (Table 2). The aerobic system’s importance to soccer was previously emphasized by the significant relationships between aerobic power and competitive ranking, team level and total distance covered during the game [1,3]. In addition, Meckel et al. [19] demonstrated that the aerobic contribution to the maintenance of the soccer game’s intensity increases during the final stages of the match.

## 5. Conclusions

The present study’s findings demonstrate a significant improvement in anaerobic capabilities such as leg power, agility, and flexibility among soccer players during the preseason phase, with no further change in mid-season. Such improvements may be the result of the power training and the high-intensity game-related training, such as small-sided games and friendly matches during the preseason phase. The improvement in aerobic fitness in the preseason is probably related to the high volume of endurance-type training during the preseason. The improvement in aerobic fitness may also be a factor in the improvement in the RST PD, leading to faster recovery between runs during the RST in the post-preseason and in-season compared to the preseason. The significant reduction in the RST IS and TS performance indices may be the result of the prolonged low-intensity rather than speed training, and possible fatigue symptoms due to the high training volume during the preseason phase. Changes in RST performance indices and their relationship to aerobic fitness throughout the soccer season should be further studied.

## Figures and Tables

**Table 1:** Changes in anthropometric and fitness variables of the players (*n* = 18) during the three stages: preseason, post-preseason and mid-season (mean ± std).

Variable	Preseason	Post-Preseason	Mid-Season
Body Mass (kg)	76.9 ± 8.4	74.7 ± 7.9 *	74.9 ± 7.6 *
Percent Fat (%)	14.6 ± 2.8	12.8 ± 2.9 *	12.6 ± 2.7 *
Flexibility (cm)	45.2 ± 8.8	48.2 ± 7.0 *	49.9 ± 6.9 *
Vertical Jump (cm)	37.0 ± 5.3	39.0 ± 4.8 *	40.3 ± 5.5 *
10 m × 4 (s)	8.1 ± 0.2	7.9 ± 0.2 *	8.1 ± 0.3

* *p* < 0.05 significant differences from preseason.

**Table 2:** A typical weekly schedule of the preseason training (upper panel) and the in-season training (lower panel).

Preseason Training							
Day	Sun.	Mon.	Tues.	Wed.	Thurs.	Fri.	Sat.
Morning	Te 30% ET 70%	Rest	FM 80% CD 20%	Rest	Te 30% ET 70%	Rest	Rest
Evening	ST 70% CD 30%	TT 30% SG 70%	Te 30% ET 70%	TT 50% ST 50%	ST 50% SG 50%	FM 80% CD 20%	Rest
**In-Season Training**							
Evening	CD 70% Te 30%	Rest	TT 40% SG 60%	ET 60% Te 40%	TT 60% SG 40%	TT 80% CD 20%	OG

Notes: ET—Endurance training; ST—Strength training; TT—Tactical training; Te—Technical training; FM—Friendly match; SG—Small-side games; OG—Official game; Cool down—CD.
